# High Moral Distress in Clinicians Involved in the Care of Undocumented Immigrants Needing Dialysis in the United States

**DOI:** 10.1089/heq.2020.0114

**Published:** 2021-07-15

**Authors:** Areeba Jawed, Sharon M. Moe, Melissa Anderson, James E. Slaven, Lucia De. Wocial, Fahad Saeed, Alexia M. Torke

**Affiliations:** ^1^ Division of Nephrology, Department of Medicine, Indiana University School of Medicine, Indianapolis, Indiana, USA.; ^2^ Department of Biostatistics, Indiana School of Medicine, Indianapolis, Indiana, USA.; ^3^ Department of Community and Health Systems, School of Nursing, Indiana University, Charles Warren Fairbanks Center for Medical Ethics IU Health, IUPUI Respect Center, Indianapolis, Indiana, USA.; ^4^ Division of Nephrology and Palliative Care, Department of Medicine, School of Public Health, University of Rochester School of Medicine and Dentistry, Rochester, New York, USA.; ^5^ Division of General Internal Medicine and Geriatrics, Indiana University School of Medicine, Indiana University Center for Aging Research, Regenstrief Institute, Inc, Indiana University Purdue University Indianapolis Research in Palliative and End-of-Life Communication and Training (RESPECT) Center, School of Nursing, Charles Warren Fairbanks Center for Medical Ethics IU Health, Indianapolis, Indiana, USA.

**Keywords:** undocumented immigrants, ethics, emergent dialysis, moral distress

## Abstract

**Purpose:** To understand clinicians' perspectives on dialysis care of undocumented immigrants.

**Methods:** A 21-item Internet-based survey using Survey Monkey^®^ was sent to 765 physicians and nurses at a safety-net hospital located in Indianapolis, IN. Moral distress thermometer score was used to assess moral distress (MD). Participants were asked to rate their MD regarding five ethically challenging clinical situations: (1) frail patients with multiple comorbidities and poor quality of life, (2) patients with dementia, (3) a noncompliant patient with frequent emergency room (ER) visits, (4) violent patients with potential harm to others, and (5) undocumented immigrants receiving emergent dialysis only.

**Key Results:** There were 299 of 775 participants (38.5% response rate) who completed the survey; 49.5% were physicians. Nearly half (48%) reported severe MD and 33% reported none to mild. In adjusted ordered logistic regression, females had significantly higher odds of MD (odds ratio [OR]=2.12, CI 1.03–4.33), and nurses had lower MD than fellows/residents (OR=0.14, CI 0.03–0.63). Over 70% of respondents attributed their distress to suffering of patients due to inadequate dialysis and tension between what is considered ethical and the law allows or forbids; 78% believed the patients' quality of life to be worse than those who receive routine hemodialysis. Among nephrologists, caring for these patients led to MD levels like that of dealing with a violent dialysis patient.

**Conclusions:** Emergent-only dialysis causes significant MD in clinicians. Legal and fiscal policies need to be balanced with the ethical and moral commitments of providers for ensuring standard of care to all.

## Introduction

“Undocumented Immigrants” are foreign born individuals who reside in the United States without appropriate legal documentation and are estimated to represent 4% of the current population,^1^ of whom ∼6,500 suffer from end-stage renal disease (ESRD).^2^ The diagnosis of ESRD grants near universal coverage for provision of dialysis to the U.S. citizens; however, undocumented immigrants are ineligible for government programs such as Medicare and nonemergency Medicaid.^3^ Approximately 65% of nephrologists in academic and private settings have reported providing dialysis to undocumented immigrants with uncertain reimbursement structures.^4^

Currently, there is considerable inconsistency in federal and state laws regarding provision of health care services to undocumented immigrants with ESRD in the United States. In 1972, an amendment to the Social Security Act guaranteed access to renal replacement therapy for all citizens with ESRD who had contributed to social security^5^; however undocumented immigrants were excluded. The 1986 amendment granted undocumented immigrants access to “emergent dialysis” under the Emergency Medical Treatment and Active Labor Act (EMTALA).^6^ Undocumented immigrants are also excluded from the patient Protection and Affordable Care Act.

Undocumented immigrants' lack of eligibility for Medicare and most forms of Medicaid presents a barrier to receiving standard care for ESRD, at least thrice weekly hemodialysis. There is considerable variation in treatment approaches, where some patients receive three times per week dialysis, while others receive emergency-only dialysis in emergency departments.^4^ Such an approach to care is not ideal and is associated with worse health outcomes including early mortality.^7–9^

Divergent practice patterns, an inability to provide the standard of care to these patients, and uncertain payment structures are likely to be a source of moral distress for clinicians. The current health care system in the United States places health care providers individually and collectively in a difficult situation in which their ethical obligation to care for all patients cannot be fulfilled within the current system due to current health care finance policies. Moral distress originally defined in the nursing literature is knowing the morally right thing to do but being unable to do it due to external constraints.^10^ One study from North Carolina conducted in free standing dialysis facilities revealed that dialyzing uninsured or undocumented residents is an ethically challenging situation experienced by 48.1% of nurses and 50% of physicians and was among the top four ethically challenging scenarios encountered by providers.^11^ In a recent study utilizing qualitative interviews in clinicians providing emergent dialysis to undocumented immigrants, four major themes were identified that included factors promoting professional burnout, moral distress from promoting injustice, confusing and perverse financial incentives, and inspiration toward advocacy.^12^

Based on prior literature, we hypothesized that moral distress would be high in providers involved in the care of undocumented immigrants receiving emergency dialysis. We conducted a survey study to gain insights from providers caring for undocumented immigrants needing dialysis at a safety net hospital in Indianapolis, Indiana, that provides emergency Medicaid coverage for emergency dialysis only.

## Methods

### Study population and setting

Between March and April 2016, we conducted an electronic survey through Survey Monkey^®^ of attending physicians, fellows, residents, and nurses at a safety net hospital providing emergency dialysis care to undocumented immigrants. The study was approved by the institutional review board of the Indiana University and Eskenazi Health. Multiple specialties and disciplines were approached including nephrology, internal medicine, critical care, palliative care, and emergency medicine. E-mail server lists were obtained from respective departments and the total number of eligible participants was 775.

### Survey design

The cross-sectional survey was developed by the authors (A.J., S.M.M., and A.M.T.). Clinical bioethicists from various specialties then pilot tested the survey, and their feedback was incorporated. To compare moral distress associated with taking care of undocumented immigrants with other potentially morally distressing situations, we adapted the moral distress thermometer^13^ as a tool for clinicians to rate the moral distress they experience in each of five ethically challenging patient situations faced in dialysis care: (1) care of undocumented immigrants receiving emergent dialysis, (2) frail patients with poor quality of life on chronic dialysis, (3) chronic dialysis patients with dementia who are unable to participate in their care, (4) noncompliant dialysis patients who make frequent emergency room (ER) visits, and (5) violent patients on chronic dialysis with potential to harm others. The latter four situations were identified as commonly occurring scenarios in dialysis care based on prior published literature.^14,15^ Dialysis for patients with poor quality of life or dementia raises questions about burdens and benefits of dialysis, whereas noncompliance and violent patients raises questions about unnecessary use of resources and the well-being of clinicians. Participants indicated the degree of distress caused by each situation on a 1–10 scale. Further questions explored dialysis care for undocumented immigrants and included contributors to moral distress, current practice patterns, perceptions regarding access to health care, and quality of life of undocumented immigrants needing dialysis. Demographic information of all participants was obtained. The survey is available online as supplementary material.

### Survey administration

An e-mail containing a survey link was sent to all potential participants introducing the purpose of the study, its voluntary nature, and the risks and benefits of participation. This was followed by two subsequent reminder e-mails every two weeks for a total of three e-mails. The survey link was not unique; however, only one survey response was accepted per electronic device. The survey was closed eight weeks after the initial e-mail was sent.

### Statistical analysis

Descriptive statistics were used to compile demographic characteristics of participants. Frequencies with percentages and means±standard deviations were calculated where applicable. Mixed effects models were employed to determine which of the ethically challenging situations caused higher moral distress to all respondents and to nephrologists alone and then with regard to how the different specialties compare with respect to the question regarding emergent dialysis in undocumented immigrants. When comparing moral distress across groups, mixed linear analysis of variance models were used, with pairwise *post hoc* comparisons using Tukey's adjustment to control for type I error. Generalized estimating equations (GEEs) were used to conduct ordinal logistic regression modeling, to identify predictors of higher moral distress. For this analysis, the dependent variable used in bivariate and multivariate analyses was a categorized score on the moral distress thermometer for the five ethically challenging situations in question. Based on the distribution of moral distress scores, the scale was divided into three categories corresponding to mild (1–3), moderate (4–6), and severe (7–10). These GEE ordinal logistic regression models were used to aid in the selection of variables for the final models based on their associations with moral distress. The variables used in both bivariate and multivariable models were chosen for clinical/conceptual reasons by the investigators and thus all were included: age, gender, ethnicity, race, current role, specialty, number of patient encounters, education in bioethics, feelings on immigrant access to adequate health care, perception of immigrant quality of life, and number of years in practice.

All analytic assumptions were verified, including the appropriateness of proportional odds, given that we are using ordinal regression models. If data appeared to be nonlinear, nonparametric tests were used to validate parametric test results. All analyses were performed using SAS v9.4 (SAS Institute, Cary, NC).

## Results

### Survey response and respondent characteristics

Out of 775 approached, 299 participants responded to the survey, corresponding to an overall response rate of 38.5%. Of returned surveys, 31 were missing demographic information and were excluded from further analysis. Respondents were 63.3% female. Of the respondents, 49.1% were nurses, 22.3% attending physicians, and the rest residents or fellows (Table 1).

**Table 1.** Characteristics of Respondents

Age (mean±standard deviation)	38.8 years ±11.2
Gender	
Male	98 (36.7%)
Female	169 (63.3%)
Ethnicity	
Hispanic or Latino	9 (3.4%)
Non-Hispanic	255 (96.6%)
Race	
White	215 (82.4%)
Black	10 (3.8%)
Asian	21 (8.1%)
American Indian	2 (0.8%)
Pacific Islander	1 (0.4%)
Other	13 (4.6%)
Current role	
Attending	60 (22.3%)
Fellow	12 (4.5%)
Resident	61 (22.7%)
Nurse	132 (49.1%)
Other	4 (1.5%)
Specialty (physicians and nurses)	
Nephrology	22 (8.2%)
Internal medicine	74 (27.7%)
Emergency medicine	70 (26.2%)
Critical care	60 (22.5%)
Palliative care	6 (2.3%)
Other	35 (13.1%)
Years in practice for nontrainees^a^	
<5 years	53 (21.0%)
5–9 years	38 (15.0%)
10–14 years	31 (12.3%)
15–19 years	20 (7.9%)
>20 years	51 (20.2%)
N/A (trainee)	60 (23.7%)
Education in bioethics	
Yes	129 (48.1%)
No	139 (51.9%)
No. of patient encounters with undocumented immigrants needing emergent dialysis	
<5	45 (16.0%)
5–10	62 (22.1%)
11–19	39 (13.9%)
>20	135 (48.0%)

Reported as number (%) unless otherwise noted. Totals do not sum to 268 due to missing data for some respondents.

^a^ Attending physicians and nurses.

### Moral distress reported in five ethically challenging situations related to dialysis therapy

For all respondents, there were significant differences in responses to the five ethically challenging situations (*F*=12.61, *p*<0.001; Fig. 2b). Results from pairwise comparisons showed that providing dialysis therapy to a violent patient caused significantly higher distress than each of the other scenarios (*p*<0.05 for all comparisons) among the situations listed. Providing dialysis to undocumented immigrants was significantly lower than to violent patients (*t*=−3.86, *p*=0.0002), significantly higher than frail (*t*=−3.25, *p*=0.0012), and similar to the other scenarios. Among the subgroup of nephrologists, there were significant differences among the scenarios for nephrologists compared with other groups (*f*=3.85, *p*<0.0058). Specifically, for nephrologists, the undocumented immigrants receiving emergent dialysis scenario caused higher moral distress than noncompliant patients' scenario (*t*=−2.81, *p*=0.0038) that was similar to violent patients (*t*=0.01, *p*=1.0000).

### Distribution of moral distress scores

**FIG. 1.**
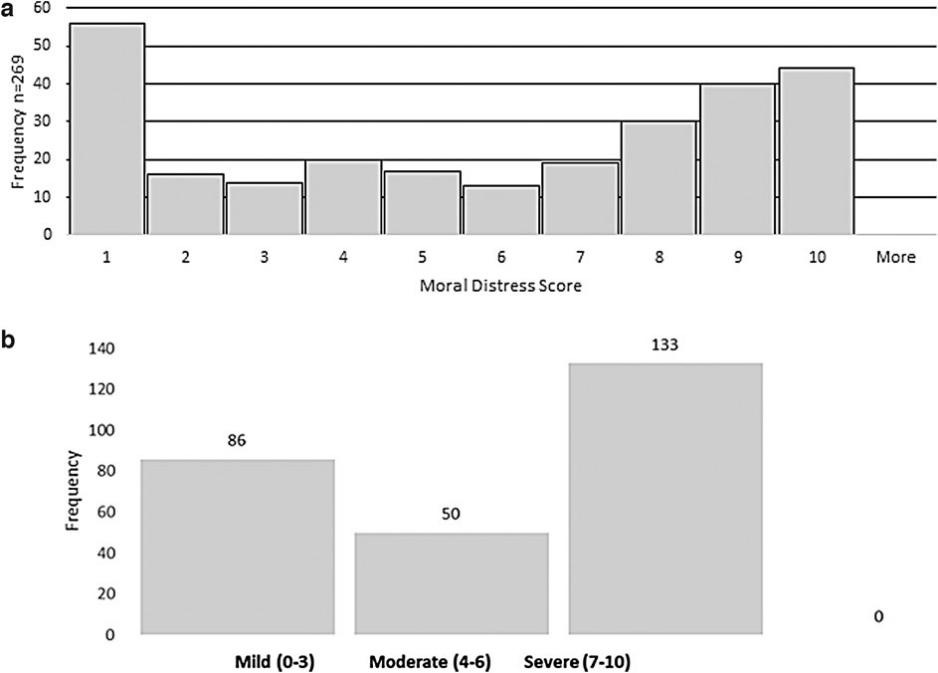
**(a)** Moral distress score experienced by entire cohort when providing emergent dialysis to undocumented immigrants. **(b)** Moral distress score experienced by entire cohort based on severity when providing emergent dialysis to undocumented immigrants.

Histogram plots for the entire cohort showed a U-shaped distribution (Fig. 1a). When results were recategorized into high, medium, and low distress, half of responders fell in the high/severe category followed by 31.9% in the mild/low category (Fig. 1b). This distribution was observed among all specialties.

### Moral distress reported in different specialties as related to providing care to undocumented immigrants receiving emergent dialysis

**FIG. 2.**
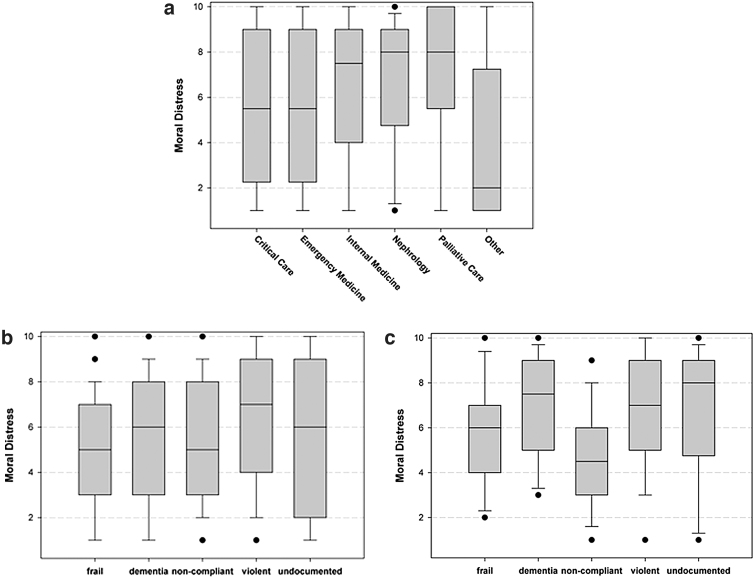
**(a)** Comparison of specialties with regard to moral distress experienced when providing care to undocumented immigrants needing emergent dialysis. **(b)** Moral distress reported in five ethically challenging situations related to dialysis therapy by entire cohort. **(c)** Moral distress reported in five ethically challenging situations related to dialysis therapy by nephrologists.

The degree of moral distress in response to dialysis for undocumented immigrants varied by specialty (*F*=4.16, *p*=0.0012; Fig. 2a). In pairwise comparisons, those who identified as “other” specialty had less moral distress than critical care (*t*=2.58, *p*=0.0105), emergency medicine (*t*=1.99 *p*=0.0480), nephrology (*t*=−3.24, *p*=0.0013), palliative medicine (*t*=−2.36 *p*=0.0191), and internal medicine (*t*=3.95 *p*<0.0001). When the data were further explored for which clinicians constituted “others,” 32 of 35 were found to be Medical-Surgical nurses. Internal medicine was also significantly higher than emergency medicine (*t*=−2.41, *p*=0.0168).

### Factors contributing to moral distress in the care of undocumented immigrants needing dialysis

When participants were asked to select from a list of potential factors contributing to the moral distress experienced in provision of care to undocumented immigrants needing dialysis, the most commonly cited factor was suffering of patients due to inadequate dialysis treatment (Table 2).

**Table 2.** Factors Contributing to Moral Distress in the Care of Undocumented Immigrants Needing Dialysis (*n*=284)

	Agree/strongly agree
Suffering of patients due to inadequate dialysis treatment	207 (72.89%)
Tension between what is considered ethical and what the law allows or forbids	198 (70.21%)
Inability to act in the best interest of the patient	181 (66%)
Compromising care due to pressure to reduce costs	158 (55.63%)
Inability to fulfill your role as patient advocate	151 (53.17%)
Lack of guidelines on how to manage patients routinely getting “emergent” dialysis	140 (49.65%)

### Perceptions regarding ESRD and care of undocumented immigrants

We found that 82% of the respondents believed the standard of care for ESRD is outpatient three times per week dialysis (Table 3). Nearly 73% of respondents disagreed with the statement that undocumented immigrants with ESRD have access to adequate health care in their state, and 78% believed that undocumented immigrants with ESRD have a quality of life that is lower than the average citizen with ESRD.

**Table 3.** Perceptions Regarding End-Stage Renal Disease and Health Care of Undocumented Immigrants

Item	Number (%)
1. Undocumented patients with ESRD have access to adequate health care in my state (*N*=281)	
Strongly agree	23 (8.19%)
Agree	25 (8.90%)
Neither	31 (11.03%)
Disagree	109 (38.79%)
Strongly disagree	93 (33.10%)
2. What do you consider to be adequate care for patients with ESRD (Please check all that apply)? (*N*=281)	
Outpatient dialysis three times per week	230 (81.85%)
Outpatient dialysis less than three times per week	41 (14.59%)
Peritoneal dialysis	98 (34.88%)
Emergent dialysis in the hospital	64 (22.78%)
Emergent dialysis in the ER	49 (17.44%)
Do not know	27 (9.61%)
3. What is your perception of the quality of life of the undocumented immigrants at your facility? (*N*=280)	
Less than the average ESRD patient receiving maintenance outpatient hemodialysis	216 (77.14%)
About the same as the average ESRD patient receiving maintenance outpatient hemodialysis	34 (12.14%)
Better than the average ESRD patient receiving maintenance outpatient hemodialysis	13 (4.64%)
Do not know	17 (6.07%)

ER, emergency room; ESRD, end-stage renal disease.

### Factors contributing to moral distress

In adjusted analysis, females had significantly higher odds of moral distress (odds ratio [OR]=2.12, CI 1.03–4.33), fellows/residents had higher moral distress than nurses (OR=0.14, CI 0.03–0.63). The gender difference in moral distress experienced was most pronounced among male and female attending physicians and less among trainee physicians (Table 4).

**Table 4.** Statistical Analysis (Unadjusted and Final Adjusted Models)

Variable	Number (%)	Median score on MD thermometer	Unadjusted comparisons			Adjusted model		
			Odds ratio	CI	*p*	Odds ratio	CI	*p*
Age	38.9 years ±11.22		0.98	[0.96–1.00]	0.11	0.99	[0.94–1.04]	0.7
Gender								
Male (ref)	97 (36.7%)	6	1.00	[0.63–1.61]	0.984	2.12	[1.03–4.33]	0.04
Female	167 (63.3%)	6						
Ethnicity								
Hispanic or Latino	9 (3.5%)	8 (10.1)	0.90	[0.23–3.14]	0.87	0.84	[0.12–5.69]	0.86
Non-Hispanic (ref)	252 (96.5%)	6 (9.2)						
Race								
White (ref)	213 (82.6%)	7						
Black	10 (3.9%)	4	0.623	[0.19–2.03]	0.98	1.29	[0.31–5.33]	0.149
Asian	21 (8.1%)	6	0.766	[0.33–1.78]	0.59	0.27	[0.09–0.82]	0.14
Other (including native Hawaii, American Indian)	14 (5.4%)	2.5	0.33	[0.12–0.95]	0.13	0.28	[0.06–1.36]	0.27
Current role								
Fellow (ref)	11 (4.41%)	8						
Resident (ref)	61 (22.93%)	8						
Attending	60 (22.6%)	7	0.66	[0.33–1.29]	0.73	0.248	[0.050–1.223]	0.45
Nurse/other	134 (50.4%)	5	0.35	[0.20–0.63]	0.001	0.14	[0.03–0.63]	0.01
Specialty								
Nephrology (ref)	22 (8.3%)	8						
Internal medicine	74 (28.0%)	7.5	0.72	[0.27–1.97]	0.33	0.724	[0.21–2.55]	0.45
Emergency medicine	69 (26.1%)	5	0.30	[0.11–0.82]	0.03	0.27	[0.08–0.97]	0.07
Critical care	59 (22.3%)	6	0.40	[0.15–1.10]	0.28	0.53	[0.14–2.12]	0.947
Palliative care	6 (2.3%)	8	2.00	[0.21–18.88]	0.14	1.2	[0.07–20.01]	0.49
Other (Medical-Surgical nurses)	34 (12.9%)	2	0.155	[0.05–0.47]	<0.001	0.22	[0.05–0.97]	0.04
No. of patients encounter								
<5 (ref)	45 (16.2%)	7						
5–10	61 (22%)	5	0.72	[0.34–1.49]	0.763	0.46	[0.166.1.295]	0.18
11–19	38 (13.7%)	5	0.61	[0.27–1.39]	0.379	0.466	[0.154–1.415]	0.25
>20	134 (48.2%)	6.5	0.77	[0.41–1.48]	0.943	0.95	[0.36–2.478]	0.17
Education in bioethics								
No (ref)	13 (51.7%)	6	1.13	[0.717–1.784]	0.597	0.918 (0.518–1.628); *p*=0.770		
Yes	128 (48.3%)	7						
Q4: Access to adequate health care by undocumented immigrants								
Disagree/neither (ref)	233 (82.9%)	6						
Agree	48 (17.1%)	6.5	1.03	[0.58–1.86]	0.910	1.33	[0.55–3.23]	0.53
Q8: QOL of undocumented immigrant compared with citizens with ESRD								
Less than (ref)	214 (77.3%)	6						
About the same	34 (12.3%)	5.5	0.99	[0.50–1.95]	0.7	0.91	[0.34–2.42]	0.40
Better than	13 (4.7%)	8	2.77	[0.82–9.4]	0.06	3.09	[0.68–14.15]	0.10
Do not know	16 (5.8%)	4	0.57	[0.22–1.47]	0.08	0.85	[0.26–2.77]	0.41
Practice years								
<5 (ref)	51 (20.4%)	5						
5–9	38 (15.2%)	5	1.04	[0.48–2.28]	0.245	1.08	[0.40– 2.86]	0.95
10–14	31 (12.4%)	6	1.55	[0.67–3.59]	0.77	1.25	[0.42– 3.74]	0.63
15–19	20 (8%)	5	1.54	[0.58–4.07]	0.83	1.23	[0.32– 4.70]	0.73
>20	51 (20.4%)	6	1.39	[0.67–2.88]	0.93	1.30	[0.31– 5.44]	0.66
N/A, trainee	59 (23.6%)	8	2.39	[1.16–4.92]	0.04	0.61	[0.15– 2.44]	0.4

MD, moral distress; QOL, quality of life.

## Discussion

Emergency dialysis is known to have worse health outcomes at an additional cost to the health care system.^7,16^ First, in this survey of clinicians in a health system that provided emergency-only dialysis to undocumented immigrants, we found nearly half of the providers had high moral distress. Most providers considered it to be less distressing than providing dialysis to a violent patient but similar to the other listed ethically challenging situations. The distribution of responses suggests that providers mostly fall into two categories: those who are highly distressed by this care and those whose distress is low, with fewer in the middle. Our results also indicate that despite nephrologists being the primary caregivers in this ethically challenging situation, there is significant moral distress in clinicians and trainees from multiple disciplines as well. Furthermore, we found no correlation between the number of patient encounters with undocumented immigrants needing emergent dialysis in the last year and the level of distress experienced, perhaps indicating that moral distress can also occur as a result of limited exposure to this ethically challenging situation.

Second, we found that trainees (fellows and residents) had higher moral distress than nurses but did not differ from attending physicians. Among physicians, trainees who are often on the frontline, yet lack autonomy, are particularly at risk for experiencing moral distress related to feeling a sense of powerlessness.^17^ In a recent qualitative study, trainees were found to have significant moral distress with regard to providing futile care in the critical care unit with emerging themes of perceived powerlessness, dehumanization, and effects of hierarchy in the medical teams.^18^ Previous studies have also shown increased frequency and intensity of moral distress associated with decreased professional autonomy.^19^ It has been postulated that the effects of hierarchy on moral distress greatly affected trainee physicians.^18^. Our results, however, did not show differences between trainee and attending physicians. A possible reason might be that rules and regulations governing dialysis therapy of undocumented immigrants are beyond any clinician's control, resulting in a sense of powerlessness and loss of professional autonomy experienced by both the attending physicians and trainees.^12^ Because nurses may also feel a lack of autonomy in these clinical situations, further research is needed to identify whether lower levels of moral distress are related to better coping, different expectations of authority, or some other factor. Moral distress has been linked to a negative impact on health care professionals' professional attitudes as well as lower job satisfaction, poor satisfaction with the quality of care provided, and burnout.^20–22^ Moral distress can also lead to longer term feelings that have been labeled “moral residue,” which lingers long after a morally problematic situation has passed, resulting in a lasting and profound loss of moral identity.^23^

Third, in this survey, 73% of the respondents felt that a major contributor to their moral distress with regard to providing emergent dialysis to undocumented immigrants was the suffering of patients due to inadequate routine three times per week dialysis treatments. Studies have explored the illness experience of undocumented immigrants on emergent dialysis and found themes of profound symptom burden, near death experiences, and family and social consequences of accommodating emergent dialysis experienced by patients undergoing emergent hemodialysis, supporting the sentiments shared by the participants in our study.^9^

Fourth, 70% of respondents in this study agreed that the tension between legal ramifications and their obligation toward patients was a contributing factor to moral distress with regard to providing emergent dialysis to undocumented immigrants. The discrepancies in laws and available funding have led to widely divergent practice patterns among states in the provision of care to undocumented immigrants with ESRD. The majority of states including ours provide only emergent dialysis^4^ to undocumented immigrants with hopes to obtain some federal funding through the EMTALA, despite most studies showing increased costs and poorer clinical outcomes associated with the practice of emergent dialysis.^8,16,24^ Undocumented immigrants are eligible for thrice weekly outpatient dialysis in 12 states. This was accomplished by reinterpretation of each state's emergency Medicaid coverage such that the revised definition of an emergency medical condition allows the same funds to cover outpatient dialysis instead of inpatient admissions for emergency-only dialysis.^25^ With the current debates involving immigration laws and reforms, policy makers have postulated that funding chronic outpatient dialysis may promote immigration to such states; however, it is noteworthy that the undocumented immigrant population of California has remained stable despite the state's stance to cover outpatient dialysis for all.^26^

Our study has multiple strengths and limitations. This is one of the first few studies to look at provider perspective and explore moral distress in this ethically challenging situation. The practice of emergent dialysis is employed most often in the care of undocumented immigrants, nationally indicating that our results are generalizable. Our survey included multiple specialties as well as nurses and physicians, making our results representative of the voice of the medical community. Our results are limited by selection and information biases inherent to survey methods, and no demographics were available for nonresponders. Unfortunately, due to issues related to access to dialysis nursing staff contact information, we were unable to capture the valuable input from dialysis nurses in our study who remain on the frontline of providing emergent dialysis. In addition, people who felt more strongly about this issue were more likely to respond given that participants were told the focus of the study was on undocumented immigrants, possibly introducing bias. Lastly this was a single center study and the survey tool had not been previously validated to answer the research question.

Our study has several implications. At the fellowship level, efforts to recognize and address moral distress could include open conversations identifying ethical challenges such as Schwartz rounds,^27^ along with training in stress management and communication skills to promote physician and nursing staff wellness.^28^ At the organizational level, system-wide efforts may include a moral distress service to help physicians and nurses resolve ethical dilemmas.^29^ Finally, at the policy level, law makers must debate the implications of emergent dialysis on providers and patients. Clinical outcomes for undocumented immigrants receiving emergent dialysis show higher mortality, increased length of stay, and poorer quality of life,^24^ whereas clinicians describe emotional exhaustion, moral distress, and burnout associated with providing substandard care.^12^

In conclusion, our findings call for a greater attention to the phenomenon of moral distress experienced by clinicians when caring for undocumented immigrants receiving emergent dialysis. It highlights the importance of balancing legal and fiscal policies with the strong ethical and moral commitments clinicians feel for ensuring safe and adequate care to all patients.

## Supplementary Material

Supplemental data

## Abbreviations Used

EMTALA: Emergency Medical Treatment and Active Labor Act
ESRD: end-stage renal disease
GEEs: generalized estimating equations
MD: moral distress
OR: odds ratio
QOL: quality of life
