# Supplementary material for: High Moral Distress in Clinicians Involved in the Care of Undocumented Immigrants Needing Dialysis in the United States
Source: Health Equity. 2021 Jul 15;5(1):484–92. doi: 10.1089/heq.2020.0114 (PMC8309436; doi:10.1089/heq.2020.0114)
Supplement: Supplemental data [file Suppl_Data.pdf]

Undocumented Immigrants Needing Dialysis

Study Description: The following research questionnaire aims to assess to moral distress experienced by health care providers involved in the care of undocumented immigrants needing dialysis. You are invited to participate as you have been identified as one of these providers. Your participation is anonymous and entirely voluntary.

You may not benefit from participating in this study. The risks of participating in this study are that you might feel uncomfortable answering some of the questions. You do not have to answer any question that makes you feel uncomfortable. There is also a risk of loss of confidentiality.

The study is being conducted by Dr. Areeba Jawed, Dr. Sharon Moe, Dr. Melissa Anderson and Dr. Alexia Torke in the Divisions of Nephrology and Internal Medicine at Indiana University School of Medicine. For questions about the study contact the principal investigator Areeba Jawed at 317-312-1709. If you cannot reach Dr. Jawed during regular business hours (i.e., 8 a.m. to 5 p.m.), please call the IU Human Subjects Office at 317-278-3458 or 800-696-2949.

Please click "next" to begin the survey.

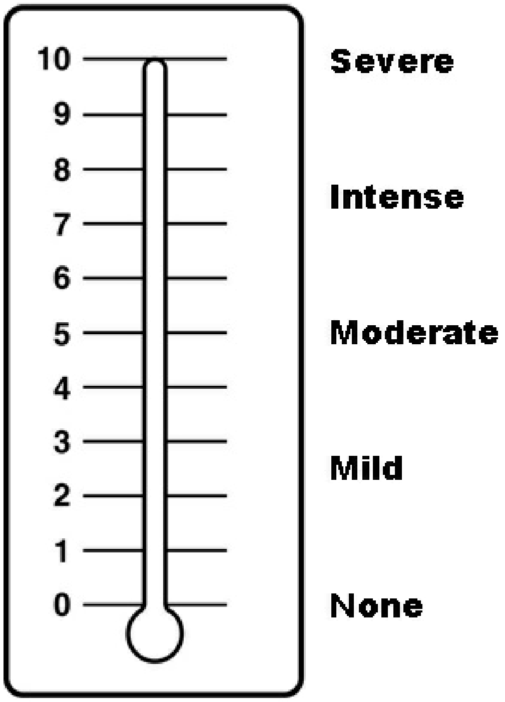

Moral Distress is a form of distress that occurs when you believe you know the ethically correct thing to do, but something or someone restricts your ability to pursue the right course of action.

1. Listed below are some ethically challenging situations encountered in patients needing<u>maintenance hemodialysis</u>. Using the Moral Distress Thermometer depicted above, please rate on a scale of 0-10 how much moral distress you would experience while participating in care of each of the following individuals.

	1	2	3	4	5	6	7	8	9	10
Providing care to a frail dialysis patient with multiple comorbidities and poor quality of life	○	○	○	○	○	○	○	○	○	○
Providing care to a dialysis patient with dementia who is unable to participate in his/her care	○	○	○	○	○	○	○	○	○	○
Providing care to a dialysis patient who is non-compliant with outpatient dialysis and makes frequent ER visits.	○	○	○	○	○	○	○	○	○	○
Providing care to a violent dialysis patient causing potential harm to providers and other patients during treatments	○	○	○	○	○	○	○	○	○	○
Providing care to an undocumented immigrant receiving emergent dialysis who is ineligible for chronic outpatient dialysis due to legal standing.	○	○	○	○	○	○	○	○	○	○

Undocumented Immigrants Needing Dialysis

2. In particular with respect to your role inproviding care to undocumented immigrants which chronic kidney disease needing renal replacement therapy please rate the following factors as contributors to the moral distress you have experienced.

	Strongly Disagree	Disagree	Neither Agree Nor Disagree	Agree	Strongly Agree
Inability to fulfill your role as patient advocate	○	○	○	○	○
Inability to act in the best interest of the patient	○	○	○	○	○
Lack of guidelines on how to manage patients routinely getting “emergent” dialysis	○	○	○	○	○
Compromising care due to pressure to reduce costs	○	○	○	○	○
Tension between what is considered ethical and what the law allows or forbids	○	○	○	○	○
Suffering of patients due to inadequate dialysis treatment	○	○	○	○	○

Undocumented Immigrants Needing Dialysis

3. In the past 12 months, approximately how many patient encounters have you had with undocumented immigrants needing dialysis?

- ◯ <5
- ◯ 5-10
- ◯ 11-19
- ◯ >20

4. Undocumented patients with end-stage renal disease (ESRD) have access to adequate health care in my state.

- ◯ Strongly Agree
- ◯ Agree
- ◯ Neither Agree Nor Disagree
- ◯ Disagree
- ◯ Strongly Disagree

5. What do you consider to be adequate care for patients with ESRD needing maintenance hemodialysis?*(Please check all that apply)*

- ☐ Outpatient dialysis 3x per week
- ☐ Outpatient dialysis less than 3x per week
- ☐ Peritoneal dialysis
- ☐ Emergent dialysis in the hospital
- ☐ Emergent dialysis in the emergency department
- ☐ Don't Know

Undocumented Immigrants Needing Dialysis

6. How is dialysis provided to undocumented ESRD patients in your practice? *(Please check all that apply)*

- ☐ Outpatient dialysis 3x per week
- ☐ Outpatient dialysis less than 3x per week
- ☐ Peritoneal dialysis
- ☐ Emergent dialysis in the hospital
- ☐ Emergent dialysis in the emergency department
- ☐ Don't Know

7. Does your institution have a written policy regarding providing dialysis to undocumented ESRD patients?

- ○ Yes
- ○ No
- ○ Don't Know

8. What is your perception of the quality of life of the undocumented immigrants at your facility?

- ○ Less than the average ESRD patient receiving maintenance outpatient hemodialysis
- ○ About the same as the average ESRD patient receiving maintenance outpatient hemodialysis
- ○ Better than the average ESRD patient receiving maintenance outpatient hemodialysis
- ○ Don’t Know

Undocumented Immigrants Needing Dialysis

9. How do you cope with the the Moral Distress that you encounter?*(Please check all that apply)*

- [ ] Venting
- [ ] Mentoring
- [ ] Team approach to patient care
- [ ] Withdrawing or detaching from distressing situation
- [ ] Nothing
- [ ] Other (please specify)

10. What do you perceive to be consequences of moral distress on you?

11. What Impact does providing care to undocumented immigrants have on you?

12. Please provide any other comments about the care of ESRD patients who are undocumented immigrants

Undocumented Immigrants Needing Dialysis

The remaining questions are included so that we will know the characteristics of responders to this survey.

13. What is your current level of training?

- ◯ Attending
- ◯ Fellow
- ◯ Resident
- ◯ Nurse
- ◯ Medical Assistant/ Technician
- ◯ Other (please specify)

14. What is your specialty?

- ◯ Internal Medicine
- ◯ Nephrology
- ◯ Emergency Medicine
- ◯ Critical Care
- ◯ Palliative Care
- ◯ Other (please specify)

15. If you are not a trainee, how long have you been in practice?

- ◯ <5 years
- ◯ 5-9 years
- ◯ 10-14 years
- ◯ 15-19 years
- ◯ 20+ years
- ◯ N/A, I am a trainee

16. What is your gender?

- Male
- Female

17. What is your year of birth? (enter 4-digit birth year; for example, 1976)

18. What is your race?

- White
- Black
- Asian
- American Indian or Alaska Native
- Native Hawaiian or other Pacific Islander
- Other (please specify)

19. In your clinical career have you received any formal education in bioethics?

- Yes
- No

20. Do you consider Nephrology to be an ethically challenging specialty?

- Yes
- No

21. What is your ethnicity?

- Hispanic or Latino
- Not Hispanic or Latino

Undocumented Immigrants Needing Dialysis

Thank You

This completes the survey. Thank you for your feedback and for your time. Please click “done” to submit your survey.
